# Supplementary material for: Copper Cobalt Sulfide Structures Derived from MOF Precursors with Enhanced Electrochemical Glucose Sensing Properties
Source: Nanomaterials (Basel). 2022 Apr 19;12(9):1394. doi: 10.3390/nano12091394 (PMC9102815; doi:10.3390/nano12091394)
Supplement: Supplementary file 1 [file nanomaterials-12-01394-s001.zip › nanomaterials-1645677-supplementary.pdf]

Supporting Information

Copper cobalt sulfide structures derived from MOF precursors with enhanced electrochemical glucose sensing properties

Daojun Zhang,[a*] Xiaobei Zhang,[a,b] Yingping Bu,[a,b] Jingchao Zhang,[a] Renchun Zhang[a]

[a] College of Chemistry and Chemical Engineering, Anyang Normal University, Anyang 455000, Henan, China

[b] College of Chemistry, Zhengzhou University, 100 Science Road, Zhengzhou 450001, Henan, P. R. China

* Corresponding author. Tel.: +86 372 2900040.

E-mail: zhangdj0410@sohu.com; zhangdj0410@126.com

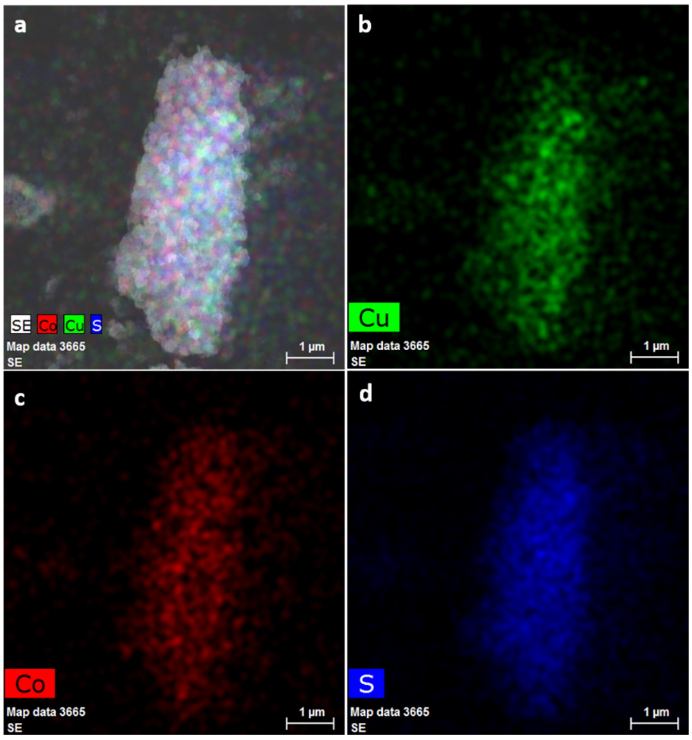

Figure S1. EDX elemental mapping images of Co-CuS-1 sample. (**a**) an overlap image of Cu, Co, and S for Co-CuS-1, elemental mapping of (**b**) Cu, (**c**) Co, and (**d**) S, respectively.

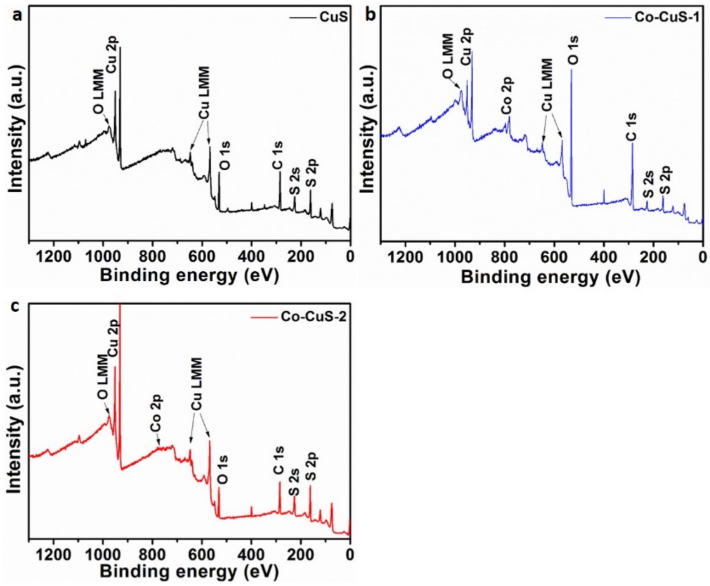

Figure S2 XPS survey spectra of (**a**) CuS-1, (**b**) Co-CuS-1 and (**c**) Co-CuS-2 samples.
